# Remission With Radiation Therapy in Primary Tongue Mucosa-Associated Lymphoid Tissue Lymphoma: A Case Report

**DOI:** 10.7759/cureus.63703

**Published:** 2024-07-02

**Authors:** Masaki Tobikawa, Jun-ichi Saitoh, Tatsuji Mizukami, Kentaro Yamagishi, Mayu Takaichi

**Affiliations:** 1 Division of Radiation Oncology, Department of Radiology, Faculty of Medicine, Academic Assembly, University of Toyama, Toyama, JPN; 2 Department of Oral and Maxillofacial Surgery, Faculty of Medicine, Academic Assembly, University of Toyama, Toyama, JPN

**Keywords:** b-cell lymphoma, non-hodgkin lymphoma, radiation therapy, tongue, mucosa-associated lymphoid tissue lymphoma

## Abstract

Mucosa-associated lymphoid tissue (MALT) lymphoma arising from the tongue is a rare pathologic condition for which a standard treatment mode has not been established. This disease represents a low-grade lymphoma frequently found in the stomach but rarely in the lymphoid tissue of the tongue. Only six cases that have been reported could be retrieved. We present the case of a 79-year-old woman who manifested with a mass on her tongue. A biopsy of the mass confirmed a diagnosis of MALT lymphoma. Radiation therapy of 30.6 Gy in 17 fractions was performed, and a complete metabolic response was achieved.

## Introduction

Malignant lymphoma is pathologically classified into non-Hodgkin’s and Hodgkin’s subtypes involving either B or T cells as origin. Their epidemiological data and clinical presentation differ significantly.

Mucosa-associated lymphoid tissue (MALT) lymphoma is categorized as non-Hodgkin’s lymphoma, and infections involving *Helicobacter pylori* and chronic inflammation processes are strongly associated with the onset and progression of MALT lymphoma [1].

MALT lymphoma is morphologically defined as a diverse mixture of cells, including centrocyte-like cells, monocytoid B-cells, small lymphocytes, and large blast-like structures, which infiltrate and proliferate mainly in the marginal zone of a follicle and between them [2].

Treatment of MALT lymphoma is distinguished with respect to nongastric and gastric subtypes. At localized stages of gastric MALT lymphoma, surgical eradication is the first choice if a test for *H. pylori* is positive; radiation therapy is the alternative if it is negative. In contrast, regarding a localized stage of nongastric MALT lymphoma, radiation therapy and surgical resection are both treatment options, although the former is more commonly used [3]. Here, we report the case of a 79-year-old woman who experienced MALT lymphoma on the tongue and underwent radiation therapy.

## Case presentation

A 79-year-old woman presented with a mass on the root of the tongue on the left side during dental caries treatment at a local dental office and was referred to our hospital for further examination and treatment. Past medical history revealed the treatment for rheumatoid arthritis with methotrexate (MTX) for over five years.

**Figure 1:**
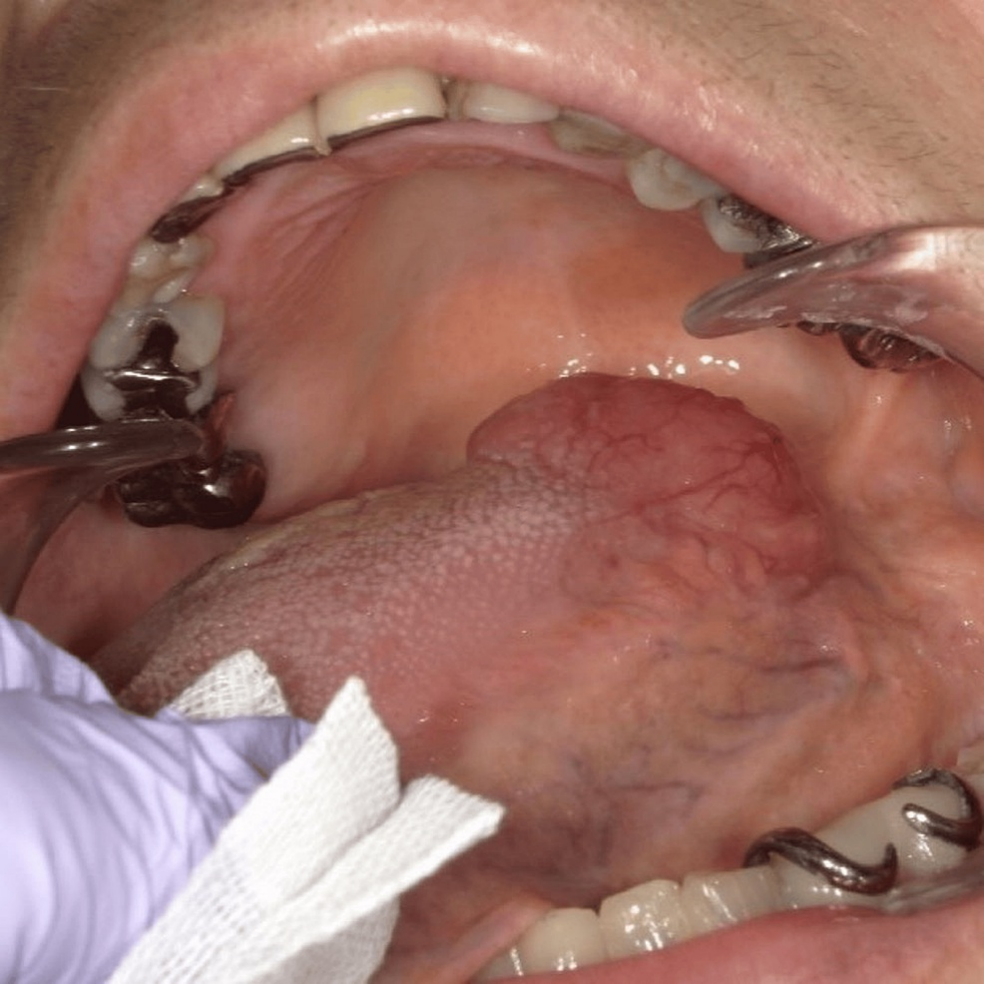
Intraoral view of the mass in the left sublingual region.

During an intraoral examination, a painless, smooth-surfaced, thin-walled, round-shaped, 20 mm in diameter, and mucosa-like color formation was observed on the root of the tongue on the left side (Figure 1).

**Figure 2:**
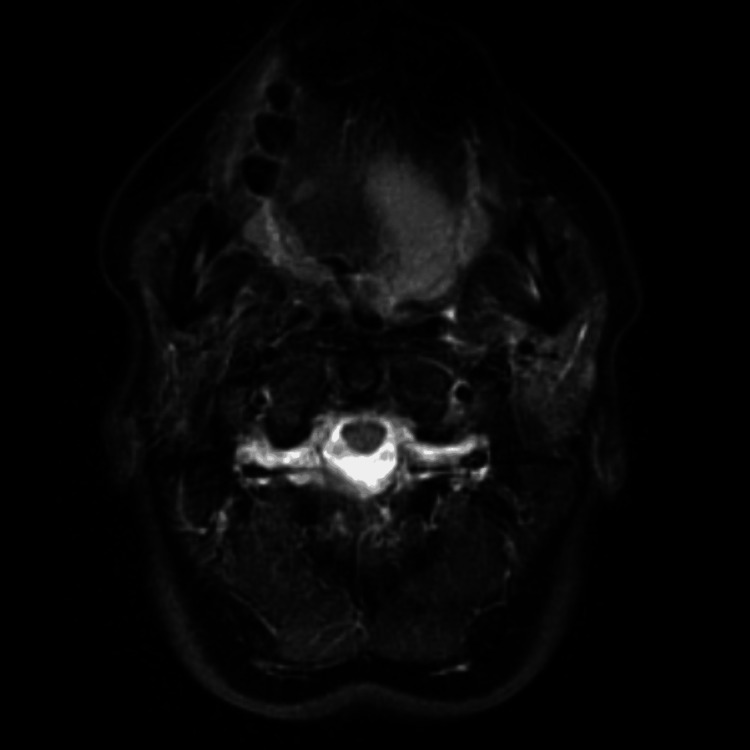
Magnetic resonance imaging short tau inversion recovery axial image showing a mass lesion with relatively uniform high signal intensity on the left lingual side of the root.

**Figure 3:**
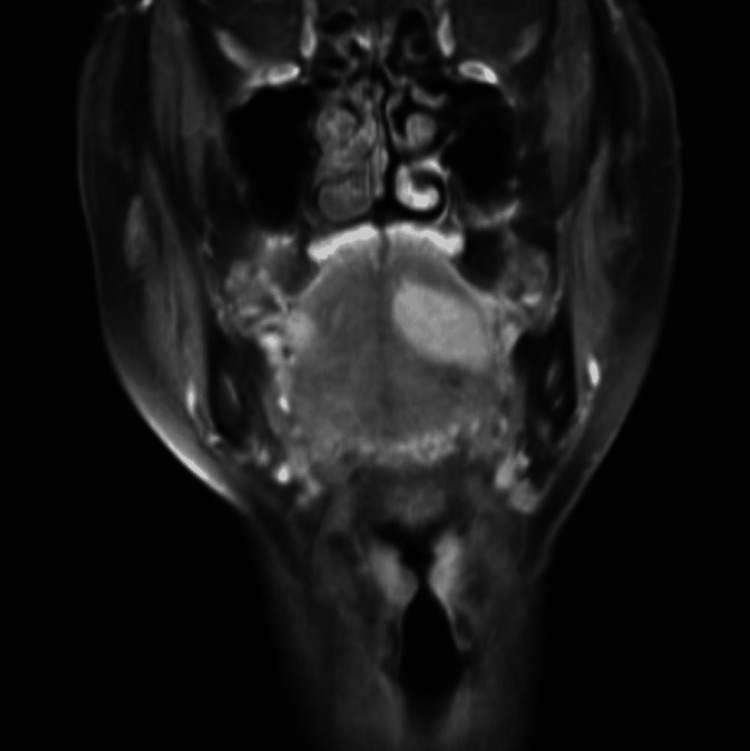
Contrast-enhanced T1-weighted coronal image with fat suppression showing a uniform contrast effect.

Magnetic resonance imaging (MRI) showed a 38 × 20 × 15 mm^3^ mass lesion on the mentioned side (Figures 2, 3).

A biopsy revealed a nodular lesion primarily in the subepithelium consisting of small B cells. Follicular colonization was observed within the nodules, with increased CD21-positive cells and tumor cells infiltrating the germinal center. B-cell infiltration was also observed between the follicles, and lymphoepithelial lesions were observed in some areas. Hence, MALT lymphoma was diagnosed.

**Figure 4:**
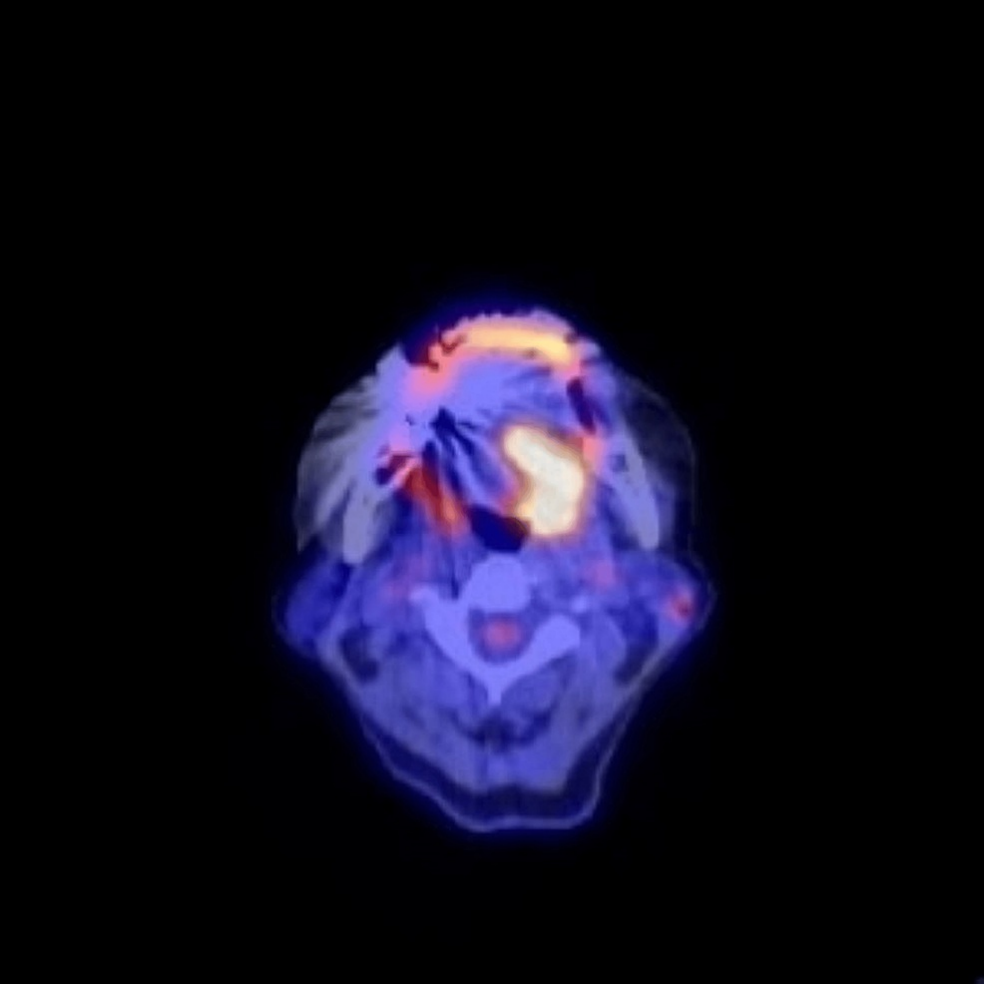
Fluorodeoxyglucose-positron emission tomography/computed tomography demonstrating a highly abnormal accumulation in the mass on the root of the tongue.

Fluorodeoxyglucose (FDG)-positron emission tomography (PET)/computed tomography (CT) scans showed highly abnormal accumulation (maximum standardized uptake value = 10.55) in the mass and none in the other sites (Figure 4).

Bone marrow biopsy and upper and lower endoscopies showed no pathologic changes. Based on these data, the final diagnosis for this patient was stage I primary MALT lymphoma of the tongue.

**Figure 5:**
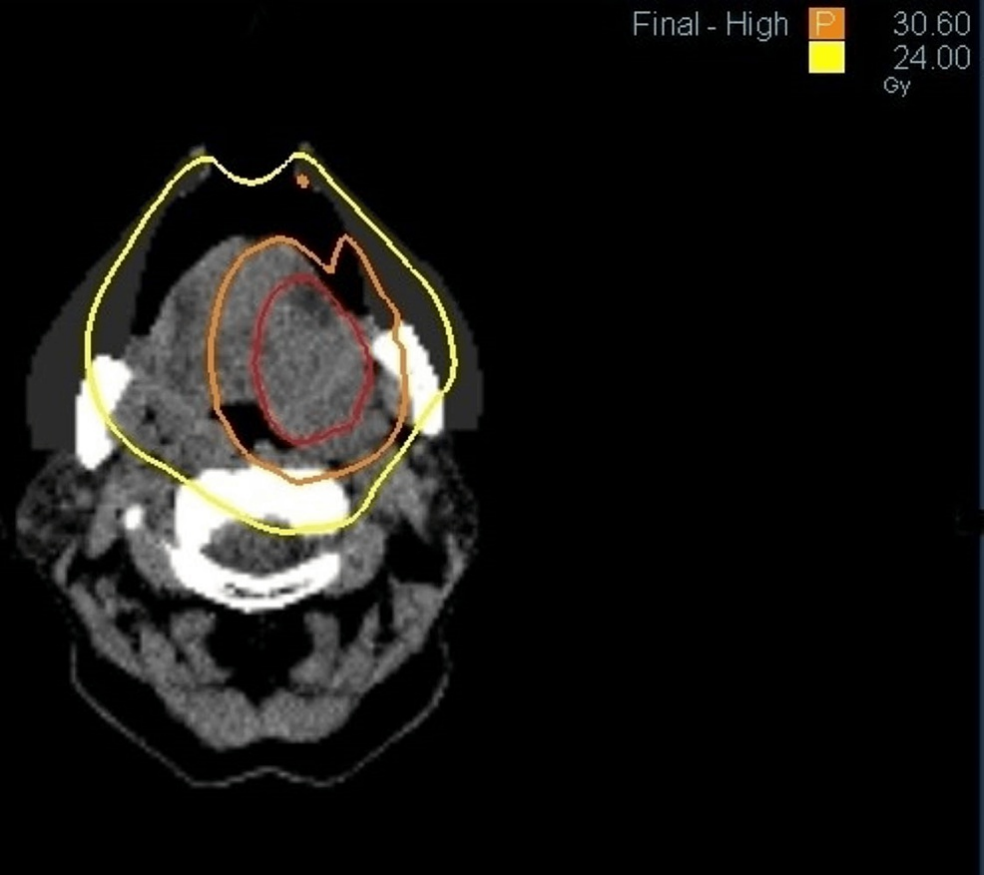
Dose distribution diagram for radiation therapy. Intensity-modulated radiation therapy using the simultaneous integrated boost method such that planning target volume 1 (gross tumor volume + 10 mm) is irradiated with 30.6 Gy and planning target volume 2 (the whole tongue + 10 mm) with 24 Gy. The orange line is 30.6 Gy, and the yellow line is 24 Gy.

Considering the possibility of lymphoma related to MTX initially taken for rheumatoid arthritis, we decided to discontinue the therapy and conducted follow-up observations. However, as the tumor continued to grow, three months after the diagnosis, we initiated radiation therapy with reference to MALT lymphoma that first appeared in the other parts of the body (Figure 5).

For radiation therapy simulation, the head and neck were fixed with a shell, and a bite block was applied. The most recent MRI short tau inversion recovery (STIR) image contoured the tumor as gross tumor volume (GTV), GTV + 10 mm was defined as planning target volume 1 (PTV1), and the whole tongue + 10 mm as PTV2, planned with intensity-modulated radiation therapy using the simultaneous integrated boost method with 30.6 Gy for PTV1 and 24 Gy for PTV2. The patient’s age and possible adverse events were significantly taken into account.

Only mild dry mouth was experienced as an acute adverse event, but the treatment was completed without serious complications. At the primary assessment six months after the end of treatment, post-treatment PET-CT imaging showed a complete metabolic response.

## Discussion

MALT lymphoma frequently develops in the stomach, appendages, salivary glands, lungs, and thyroid glands, but in the tongue, it is very rare. MALT lymphoma diagnosis, proposed by Isaacson et al. in 1983, is a low-grade lymphoma derived from MALT [4]. It is outlined as a lymphoma, in which morphologically diverse B cells infiltrate tissues and proliferate mainly from a follicular marginal zone to interfollicular spaces [2]. The stomach is the most common site of MALT lymphoma, accounting for about 50% of the total cases.

MALT lymphoma is frequently associated with infection and chronic inflammation. Concerning the former, *H. pylori* is linked to the development of MALT lymphoma of the stomach, *Chlamydophila psittaci* to ocular adnexa,* Campylobacter jejuni* to the small intestine, and *Borrelia burgdorferi* to the skin [5-7]. Regarding chronic inflammatory diseases, Sjögren syndrome is connected with the development of MALT lymphoma of salivary glands and chronic thyroiditis [8,9]. In this case, the patient had been treated for rheumatoid arthritis for a long time, but the site of onset was the tongue, indicating a possibly low association.

The patient had been administered MTX for rheumatoid arthritis, raising considerations of MTX-associated lymphoproliferative disorders (MTX-LPDs). MTX-LPD is a disease in which lymphoproliferative processes typical of lymphoma occur in patients undergoing MTX therapy for a long time [10]. MTX-LPD is characterized by a female predilection, more extranodal lesions, and a greater proportion of Epstein-Barr virus-positive cases. Regarding histopathological types of MTX-LPD, diffuse large B-cell lymphoma is the most commonly detected, followed by Hodgkin’s lymphoma [11]. In this case, who had been receiving MTX for at least five years, MTX-LPD was considered. However, given that MALT lymphoma rarely develops from MTX-LPD and no reduction in tumor size was observed after the withdrawal of the drug, the association may be low.

Treatment strategies for MALT lymphoma vary based on the location. For MALT lymphoma that develops in areas other than the stomach, stage I disease is primarily managed with radiation therapy, surgical resection, or no treatment. Stages II to IV MALT lymphoma is mainly treated with rituximab combination chemotherapy.

The case of MALT lymphoma confined to the tongue is uncommon. We searched the literature for MALT lymphoma arising from the tongue and identified only six relevant reports (Table 1). Four of seven patients (including our case) were women. Regarding the medical history, it was described in five cases involving the following conditions: T-cell lymphoma, Sjögren syndrome, rheumatoid arthritis, and two cases of no history. The average patient age was 54 years (range = 18-80 years). Treatment options included surgical excision in four cases, radiation therapy in two, and chemotherapy in one, but none of them showed relapse during follow-up [12-17].

**Table 1:** Case reports of MALT lymphoma of the tongue. MALT = mucosa-associated lymphoid tissue; M = male; F = female

Authors	Age	Sex	History	Treatment	Clinical course
Kuramoto et al. (1995) [12]	43	M	Unknown	Surgical excision	No recurrence 6 months after treatment
Goteri et al. (2004) [13]	80	F	No history	Surgical excision	Confirmed alive 1 year after diagnosis
Song et al. (2014) [14]	29	F	T-cell lymphoma	Radiation therapy (30.6 Gy/17 Fr)	No recurrence 3 years after treatment
Iftikhar et al. (2016) [15]	61	M	Unknown	CHOP 8 cycles	No recurrence 19 months after treatment
Lyapichev et al. (2022) [16]	69	F	No history	Surgical excision	No recurrence 10 years after treatment
Aleksiejūnaitė et al. (2023) [17]	18	M	Sjögren syndrome, ranula	Surgical excision	No recurrence 5 months after treatment
Tobikawa et al. (2023) (current study)	79	F	Rheumatoid arthritis	Radiation therapy (30.6 Gy/17 Fr)	No recurrence 9 months after treatment

## Conclusions

Extranodal marginal zone B-cell MALT lymphoma of the tongue is a rare condition. For localized MALT lymphomas, surgery is also considered a treatment option. In this case, surgical resection or radiotherapy was considered. However, due to the location of the tumor at the root of the tongue, more complications were predicted for the resection, thus radiation therapy was ultimately preferred. This treatment led to a complete metabolic response, confirming the effectiveness of radiation therapy for MALT lymphoma at the tongue as well as at other sites.
